# Extracellular vesicle-coupled miRNA profiles of chicken seminal plasma and their potential interaction with recipient cells

**DOI:** 10.1016/j.psj.2023.103099

**Published:** 2023-09-12

**Authors:** Xintong Han, Yunlei Li, Yunhe Zong, Dongli Li, Jingwei Yuan, Hanhan Yang, Hui Ma, Aixin Ni, Yuanmei Wang, Jinmeng Zhao, Jilan Chen, Tenghe Ma, Yanyan Sun

**Affiliations:** ⁎ State Key Laboratory of Animal Biotech Breeding, Key Laboratory of Animal (Poultry) Genetics Breeding and Reproduction of Ministry of Agriculture and Rural Affairs, Institute of Animal Sciences, Chinese Academy of Agricultural Sciences, Beijing, 100193, China; † College of Life Sciences and Food Engineering, Hebei University of Engineering, Handan, 056038, Hebei, China; ‡ Beijing Huadu Yukou Poultry Industry Co. Ltd., Beijing, 101206, China; § College of medicine, Hebei University of Engineering, Handan, 056000, Hebei, China

**Keywords:** chicken, seminal plasma, extracellular vesicle, miRNA

## Abstract

The presence of EVs in seminal plasma (**SPEVs**) suggests their involvement on fertility via transmitting information between the original cells and recipient cells. SPEVs-coupled miRNAs have been shown to affect sperm motility, maturation, and capacitation in mammals, but rarely in poultry species. The present study aims to reveal the profile of SPEVs miRNAs and their potential effect on sperm storage and function in poultry. The SPEVs was successfully isolated from 4 different chicken breeds by ultracentrifugation and verified. Deep sequencing of SPEVs small RNA library of each breed identified 1077 miRNAs in total and 563 shared ones. The top 10 abundant miRNAs (such as miR-10-5p, miR-100-5p, and miR-10a-5p etc.) accounted for around 60% of total SPEVs miRNA reads and are highly conserved across species, predisposing their functional significance. Target genes prediction and functional enrichment analysis indicated that the most abundantly expressed miRNAs may regulate pathways like ubiquitin-mediated proteolysis, endocytosis, mitophagy, glycosphingolipid biosynthesis, fatty acid metabolism, and fatty acid elongation. The high abundant SPEVs-coupled miRNAs were found to target 107 and 64 functionally important mRNAs in the potential recipient cells, sperm and sperm storage tubules (**SST**) cells, respectively. The pathways that enriched by target mRNAs revealed that the SPEVs-coupled miRNA may rule the fertility by affecting the sperm maturation and regulating the female's immune response and lipid metabolism. In summary, this study presents the distinctive repertoire of SPEVs-coupled miRNAs, and extends our understanding about their potential roles in sperm maturation, capacitation, storage, and fertility, and may help to develop new therapeutic strategies for male infertility and sperm storage.

## INTRODUCTION

Extracellular vesicles are bowl-shaped vesicles with a bilayer structure secreted into the extracellular system of cells. Almost all cell types especially liver cells, tumor cells, red blood cells, and epithelial cells, etc. are able to produce extracellular vesicles (Keerthikumar et al., 2015; Muroya et al., 2016). Extracellular vesicles served as a key mediator of intercellular communication and are associated with different reproductive events, including gamete maturation, fertilization, and embryo and fetal development (Du et al., 2016; Gurunathan et al., 2022).

The extracellular vesicles in seminal plasma of human (Candenas and Chianese, 2020), mouse (Wang et al., 2021), and fish (Zhang et al., 2020) were reported to be of epididymal or sexual accessory gland origin, which is underdeveloped in poultry species. However, the presence of seminal plasma extracellular vesicles (**SPEVs**) has been recently demonstrated in chickens and actively involved in sperm viability and motility regulations (Cordeiro et al., 2021a). Luo et al. (2022) detected a number of 3,073 proteins enriched to provide energy and transport proteins in extracellular vesicles of chicken seminal plasma.

Besides proteins, extracellular vesicles encapsulate a rather enriched complex load of other biological components including lipids, small non-coding and regulatory RNAs (**ncRNAs**) (Jeppesen et al., 2019). MicroRNAs (**miRNAs**) are a class of endogenous ncRNAs of 20-25 nucleotides long with regulatory functions found in eukaryotes. Extracellular vesicles miRNAs have been shown to be associated with immune function and endothelial cell proliferation (Sun et al., 2021; Yan et al., 2022). The miR-31 may play a role in regulating mitochondria during cytoplasmic droplets migration (Sun et al., 2021). The expression level of miR-184 in the seminal plasma EVs of infertility patients is significantly different from that of normal fertile males, and this miR-184 may regulate genes involved in spermatogenesis related pathways (Zhu et al., 2020).

The miR-210-3p plays a protective role under hypoxic stress through regulating cell survival, proliferation, inflammation, and metabolism (Meng et al., 2019). The hypoxia promoted miR-210-3p was carried by seminal exosomes which was released from Sertoli cells, and its expression may be associated with the function of Sertoli cells (Ma et al., 2021). However, the small RNAs profile of chicken SPEVs and their potential functions have not been investigated yet.

The transportation of sperm through vas deferens and sperm storage within the female reproductive tract is important for gaining and maintaining fertility (Orr and Zuk, 2012; Xing et al., 2022). Extracellular vesicles transport miRNAs in both paracrine (between cells of the same origin) and endocrine (to distant targets cells) ways (Yu et al., 2016b). The testis, epididymis, or vas deferens originated extracellular vesicles in the seminal plasma may play roles via interacting with the spermatozoa during the sperm transport for sperm maturation (Baskaran et al., 2020; Li et al., 2022), and even with the sperm storage tubules (**SST**) cells of uterovaginal junction during the sperm storage in females.

However, quite few studies have been focusing on this topic and limit the understanding of extracellular vesicle-coupled miRNA profiles of chicken seminal plasma. Therefore, in order to further study the effect of SPEVs miRNAs on sperm function and reproductive breeding in poultry, 4 breeds of chickens were first selected for SPEVs extraction and verification, followed by the construction of miRNA profiles of SPEVs, and the potential interaction of high-abundance miRNAs with sperm and SST mRNA was explored.

## MATERIALS AND METHODS

### Ethics Statement

All animal experiments were approved by the Animal Care and Use Committee at the IAS-CAAS (No. IAS2022-70, 2022-05-16) and conducted at the institute. All of the experiments followed relevant guidelines and regulations set by the Ministry of Agriculture and Rural Affairs of the People's Republic of China.

### Animals

Chicken of 4 breeds, including the dual-purpose Beijing-You chicken (BJY, n = 50), Dwarf chicken (DY, n = 50), and Recessive White chicken (IV, n = 50), and an elite layer breed White Leghorn (WL, n = 50) from the experimental farm of Institute of Animal Science, Chinese Academy of Agriculture Sciences (IAS-CAAS), were used in this study. Staring from 19 wk of age, they were reared in individual cages (40 cm in length, 45 cm in width, and 45 cm in height) with controlled temperature and light. Feed and water were provided ad libitum. At 50 wk of age, each 40 roosters per breed of normal semen quality (sperm motility > 0.7 and sperm concentration > 1.0 × 10^8^/mL) were identified after the estimation using the computer-aided semen analysis (CASA) system (ML-608JZII; Nanning Songjingtianlun Bio-technology Co., Ltd., Guangxi, China).

### Seminal Extracellular Vesicles Isolation

Semen for extracellular vesicles isolation were collected form the 40 chickens from each breed using the torso-abdominal massage method. The necessary care was taken to avoid any contamination with transparent fluid and other cloacal products. Semen for extracellular vesicles isolation were collected form the 40 chickens from each breed using the torso-abdominal massage method. The necessary care was taken to avoid any contamination with transparent fluid and other cloacal products. The collected semen (around 20 mL per breed) was pooled and centrifuged at 1,000× *g* at 4°C for 10 min. The precipitation was discarded and the supernatant was collected and stored on ice. Sperm pellet was gently resuspended with 500 µL phosphate buffer solution (PBS) with protease inhibitor and centrifuged as at 1,000× *g* at 4°C for 10 min. The precipitation was resuspended with PBS and centrifuged again, and this procedure was repeated 3 times. The supernatant collected by 3 centrifugations was mixed evenly and centrifuged at 20,000× *g* at 4°C for 15 min. The precipitation was discarded and the supernatant was collected after centrifugation at 100,000× *g* at 4°C for 90 min. The supernatant was discarded, and the circular white precipitate was attached to the tube wall. The precipitate was resuspended in 200 µL PBS and stored at −80°C until further manipulation.

### Identification of Seminal Extracellular Vesicles

#### Characterization by Transmission Electron Microscopy

SPEVs samples were isolated from the seminal fluid, fixed in 2% paraformaldehyde, dropped onto copper grids, and incubated without movement for 5 min. SPEVs adhered to copper grids were washed with PBS. The excess liquid was absorbed from the edge of the copper grids with filter paper, phosphotungstic acid negative staining solution was added, and the grids were again incubated without movement for 5 to 10 min. The excess staining solution was absorbed, after which the grids were allowed to dry naturally. The copper grids were placed in the sample seat for further observation with a H-7500 transmission electron microscope (Hitachi, Tokyo, Japan).

#### Nanoparticle Tracking Analysis

We measured the SPEVs particle size and concentration using nanoparticle tracking analysis (**NTA**) at VivaCell Biosceinces with ZetaView PMX 110 (Particle Metrix, Meerbusch, Germany) and the corresponding software ZetaView 8.04.02. Isolated exosome samples were appropriately diluted using 1 × PBS buffer (Biological Industries, Kibbutz Beit Haemek, Israel) before the measurement. NTA measurement was recorded and analyzed at 11 positions. The ZetaView system was calibrated using 110 nm polystyrene particles. Temperature was maintained around 26°C.

#### Western Blot Analysis

Immunoblotting was performed on 3 extracellular vesicles specific marker proteins including ALG-2 interacting protein X (**ALIX**), heat shock protein 70 (**HSP70**), and tumor susceptibility 101 (**TSG101**). After reaction on ice for 30 min with 100 uL RIPA lysate (R0020-100 mL, Solarbio, Beijing, China) added to the sample, the supernatant was collected for detection of protein concentration using the BCA protein Detection Kit (PA115-01, TIANGEN, Beijing, China) after centrifugation at 20,000× *g* at 4°C for 10 min. After the adding of 5× protein loading buffer (P1041, Solarbio), the samples were boiled at 95°C for 10 min. After gelation, 20 µg of proteins were loaded on 15% SDS-PAGE (PG114, EpiZyme, Shanghai, China) at 90 V for 90 min, after which the proteins were transferred to polyvinylidene fluoride (**PVDF**) membrane at 300 mA for 60 min. The PVDF membrane was blocked with 5% skim milk powder (232100-100, BD-Pharmingen, Franklin L., New Jersey) for 1 h. After cleaning, the primary antibodies were incubated at 4°C overnight. The 3 primary antibodies used were ALIX (A2215, 1:500, Abclonal, Wuhan, China), HSP70 (A20819, 1:2,500, Abclonal), and TSG101 (ab275018-1kit, 1:1,000, Abcam, Shanghai, China). After washing, the membranes were incubated with secondary antibody (G1213-100UL, 1:5,000, Servicebio, Wuhan, China) for 1 h at room temperature. The image of protein band signals was captured using the imaging system (Tanon-5200, Tanon, Shanghai, China) after ECL luminescence solution processing.

#### Total RNA Extraction, Library Preparation, and Sequencing

After total RNA was extracted by the Trizol reagent kit (Invitrogen, Carlsbad, CA), the RNA molecules in a size range of 18 to 30 nt were enriched by polyacrylamide gel electrophoresis (**PAGE**). Then the 3’adapters were added and the 36 to 44 nt RNAs were enriched. The 5’adapters were then ligated to the RNAs as well. The ligation products were reverse transcribed by PCR amplification and the 140 to 160 bp size PCR products were enriched to generate a cDNA library and sequenced using Illumina HiSeq 2500 by Gene Denovo Biotechnology Co. (Guangzhou, China).

#### Sequencing Data Analysis, miRNA Identification, and Target Genes Prediction

All of the clean tags were aligned with small RNAs in GeneBank database (Release 209.0) to identify and remove ribosomal RNA (**rRNA**), small cytoplasmic RNA (**scRNA**), small nucleolar RNA (**snoRNA**), small nuclear RNA (**snRNA**), and transfer RNA (**tRNA**). Meanwhile all of the clean tags were aligned with small RNAs in Rfam database (Release 11.0) to identify and remove rRNA, scRNA, snoRNA, snRNA, and tRNA. All of the clean tags were also aligned with the reference genome (Ensembl_release106). Those tags that mapped to exons or introns might be fragments from mRNA degradation, and were therefore removed. The tags mapped to repeat sequences were also removed. All of the clean tags were then searched against miRBase database (Release 22) to identify known chicken miRNAs. So far, the miRNA sequences of some species were still not included in miRBase database. For those species the miRNAs alignment with other species was a dependable way to identify the known miRNAs. All of the unannotated tags were aligned with reference genome. According to their genome positions and hairpin structures predicted by software MiReap_v0.2, the novel miRNA candidates were identified. The miRNA profile was also compared with the reported SPEVs miRNA of human, bovine and porcine (Vojtech et al., 2014; Alves et al., 2021; Sun et al., 2021). Two software MiRanda (Version 3.3a) and TargetScan (Version 7.0) were used to predict targets genes of the miRNAs. The intersection of the results was more credible to be chosen as predicted miRNA target genes.

#### Combined Analysis of SPEVs miRNA—Sperm mRNA and SPEVs miRNA-SST mRNA

The profile of sperm mRNA and SST mRNA of potential importance was exacted from the previous publication and database (SST RNA-sequencing data was uploaded to NCBI and is not yet available) (Singh et al., 2016), i.e. the differentially expressed mRNA in the sperm (between sperm and testis, *P* < 0.01, fold change > 7) and in the SST cells (between high and low sperm storage ability individuals, *P* < 0.01, fold change > 1.5). The regulatory network analysis of miRNA-mRNA was performed using the Cytoscape (v3.9.1).

#### Gene Ontology and Kyoto Encyclopedia of Genes and Genomes Enrichment Analysis

Gene ontology (**GO**) and Kyoto Encyclopedia of Genes and Genomes (**KEGG**) enrichment analysis were performed on 1) the target genes of miRNAs shared by the 4 breeds selected in order to comprehensively figure out their biological functions; 2) the target genes of the miRNAs of top 10 abundance; 3) the shared genes between targets of the SPEVs miRNA of top 10 abundance and the key sperm mRNA; and 4) the shared genes between targets of the SPEVs miRNA of top 10 abundance and the key SST mRNA. Gene ontology analysis were categorized into 3 categories, biological process, cellular component and molecular function. The GO and KEGG enrichment analysis was performed using the OmicShare tools (http://omicshare.com/tools).

## RESULTS

### SPEVs Characterization

**Figure 1:**
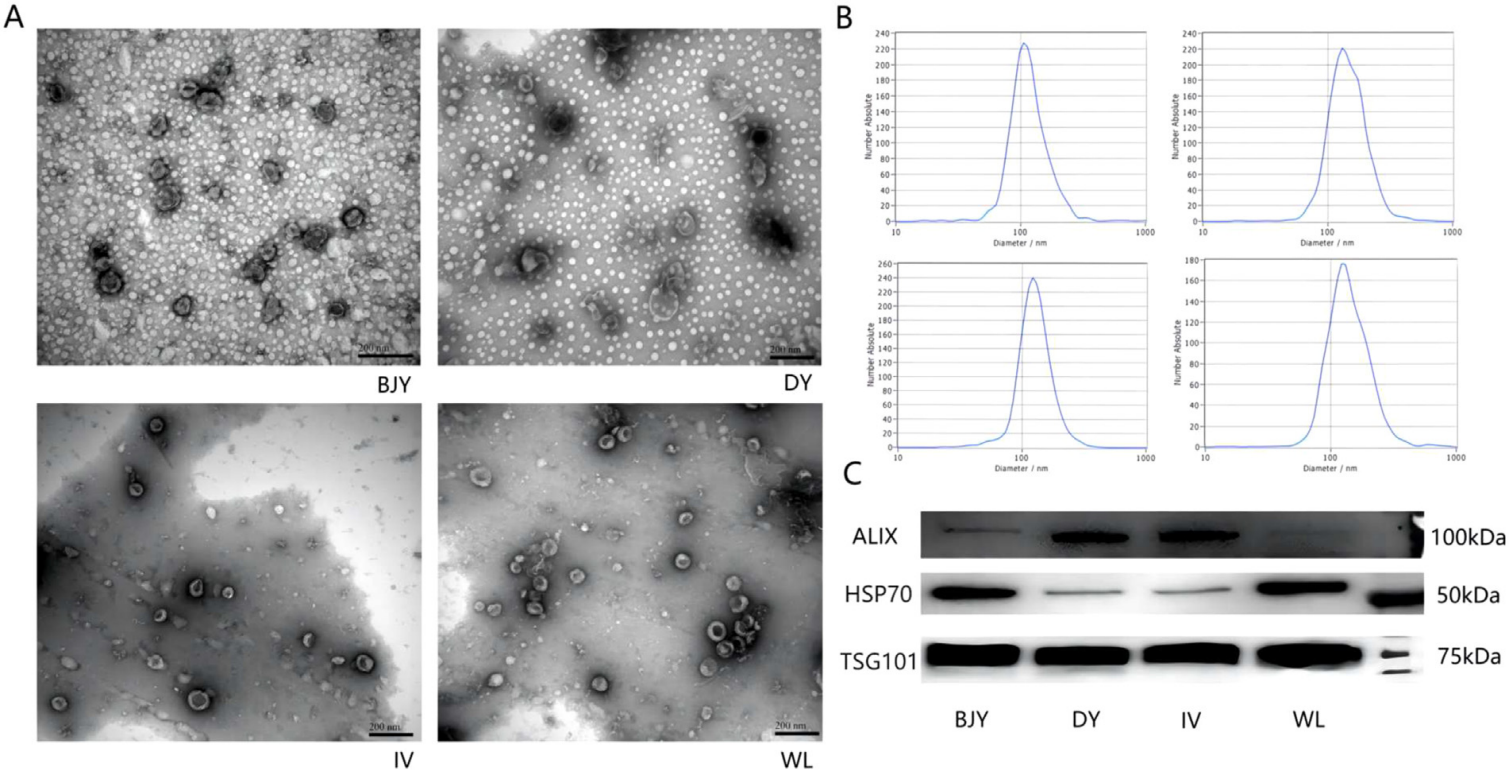
The characteristic features of chicken seminal plasma extracellular vesicles (SPEVs). (A) Transmission electron microscope images of SPEVs isolated from 4 chicken breeds, Beijing-You chicken (BJY), Dwarf chicken (DY), Recessive white chicken (IV), and White Leghorn (WL). Bar = 200 µm. SPEVs possess typical cup-shaped vesicle structures. (B) Nanoparticle tracking analysis of particle size distribution profiles of SPEVs from 4 chicken breeds. Most particle sizes ranged from 50 to 200 nm which is consistent with the characteristics of EVs. (C) Western blots analysis of ALIX, TSG101, and HSP70 proteins of SPEVs from 4 chicken breeds.

Chicken SPEVs were isolated and their morphology and structure were observed using transmission electron microscopy (**TEM**) and NTA. Transmission electron microscopy showed that SPEVs had an obvious teacup structure (Figure 1A). As shown in the NTA results (Figure 1B), the concentrations of SPEVs was 5.8 × 10^7^, 3.4 × 10^7^, 1.1 × 10^7^, and 4.9 × 10^7^ particles/mL for BJY, DY, IV, and WL, respectively; the average SPEVs diameter was 110.0, 139.3, 125.6, and 129.2 nm for BJY, DY, IV, and WL, respectively, being between 50 and 150 nm, which is in line with the standards in MIVSE2018 (Théry et al., 2018). The expression of 3 SPEVs specific protein markers ALIX, HSP70, and TSG101 were detected by Western Blot, although the abundance varied in 4 breeds (Figure 1C).

### Brief Data Presentation of Small RNA Sequencing Libraries

Approximately 4.85 million total clean reads were found, including 9907157, 12811406, 12149116, and 13667058 reads in BJY, DY, IV, and WL, respectively. Mapped RNAs were sorted by biotype. Known miRNA reads accounted for 69.92% to 77.16% of the RNA, and novel miRNA reads accounted for 0.18% to 0.23% of the RNA. Other RNA biotype including rRNA, scRNA, snoRNA, snRNA, and tRNA reads were also detected. The tRNA reads accounted for around 10% of the RNA (Table 1). We mainly focused on the miRNA data from RNA-seq analysis for further investigation in this study.

**Table 1:** Data summary of 4 small RNA sequencing libraries.

Sample	Total	Clean reads	Known miRNA	Novel miRNA	rRNA	scRNA	snoRNA	snRNA	tRNA
All	48534737	46037548	33244523	94423	1459454	302100	650	44597	4640964
			72.17%	0.21%	3.17%	0.66%	0.00%	0.10%	10.08%
BJY	9907157	9328939	7198345	19198	238109	6951	88	14176	593098
			77.16%	0.21%	2.55%	0.07%	0.00%	0.15%	6.36%
DY	12811406	12112571	7620711	24977	593537	207679	232	10963	2234971
			62.92%	0.21%	4.90%	1.71%	0.00%	0.09%	18.45%
IV	12149116	11612742	8755034	26259	369585	60762	180	9196	936999
			75.39%	0.23%	3.18%	0.52%	0.00%	0.08%	8.07%
WL	13667058	12983296	9650433	23989	258223	26708	150	10262	875896
			74.33%	0.18%	1.99%	0.21%	0.00%	0.08%	6.75%

Abbreviations: BJY, Beijing-You chicken; DY, Dwarf chicken; IV, Recessive white chicken; rRNA, ribosomal RNA; scRNA, small cytoplasmic RNA; snoRNA, small nucleolar RNA; snRNA, small nuclear RNA; tRNA, transfer RNA; WL, White Leghorn.

### Identification of miRNAs

**Figure 2:**
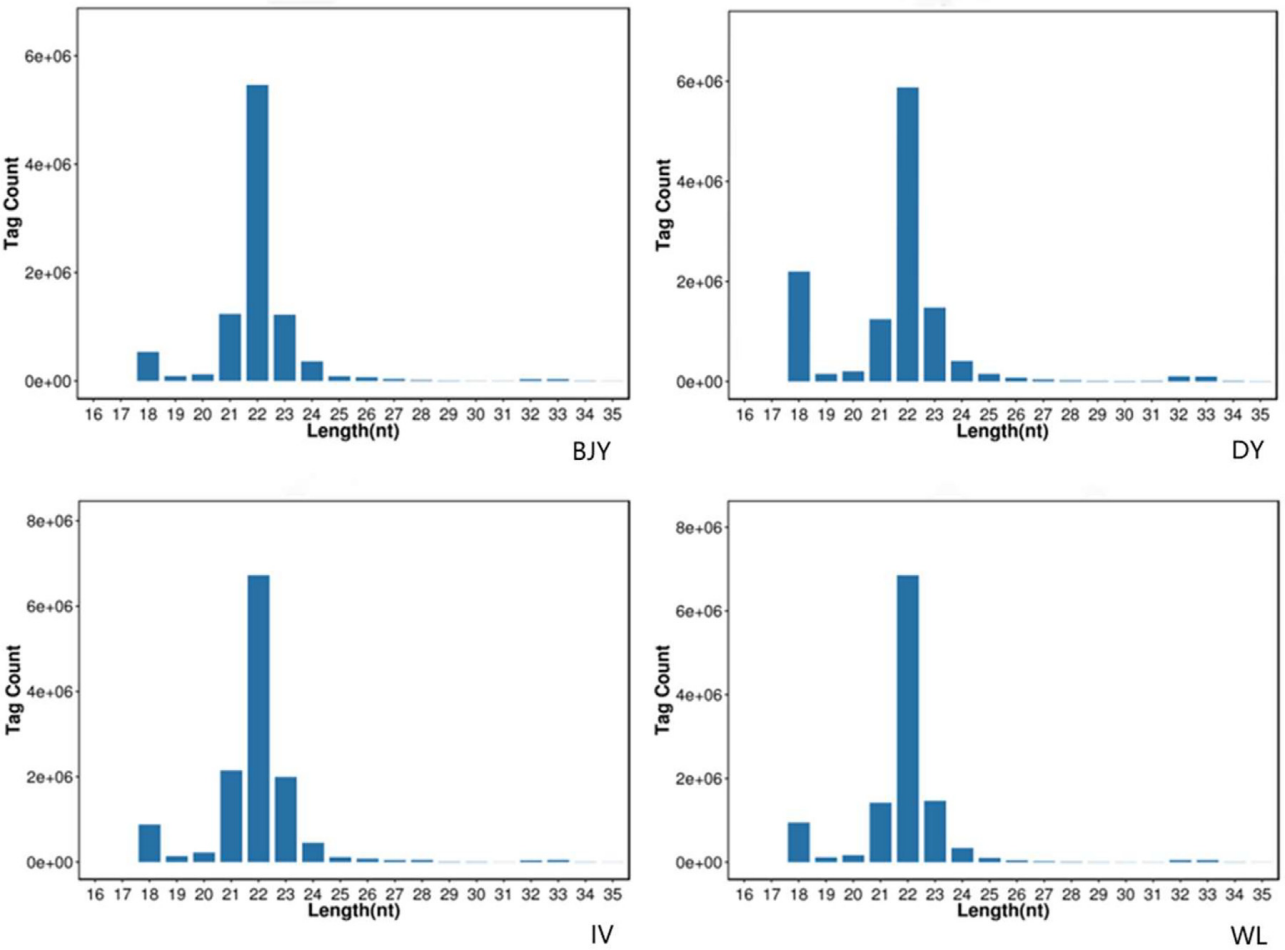
Length distribution of small RNAs sequence of chicken seminal plasma extracellular vesicles. The identified small RNAs ranged between 18 and 35 nt for 4 chicken breeds, Beijing-You chicken (BJY), Dwarf chicken (DY), Recessive white chicken (IV), and White Leghorn (WL).

**Figure 3:**
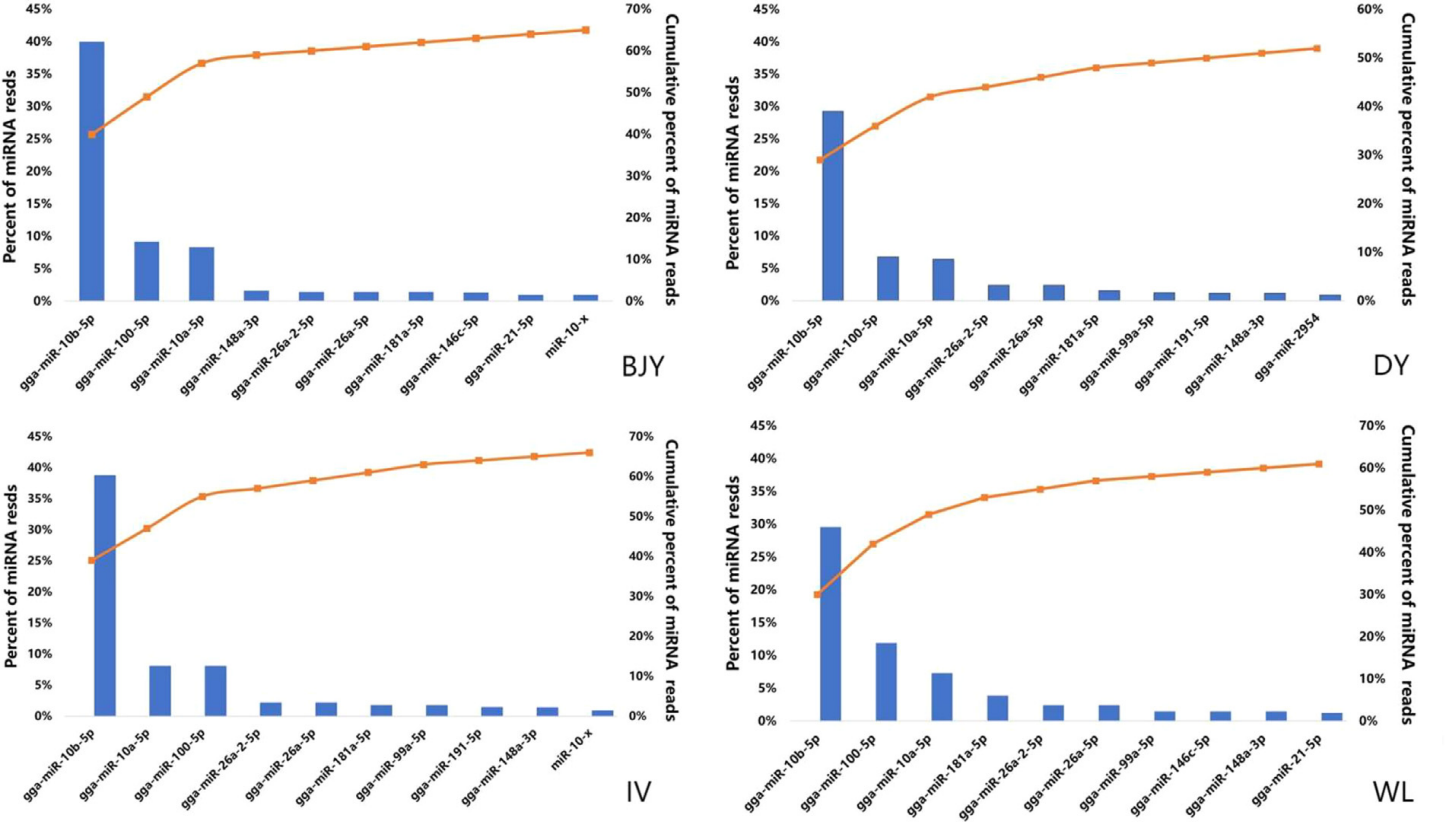
The top 10 most abundant miRNAs in each breed. Left axis and the bars: percentage of each miRNA of the total miRNA reads. Right axis and dot: cumulative percentage of miRNA reads. Abbreviations: BJY, Beijing-You chicken; DY, Dwarf chicken; IV, Recessive white chicken; WL, White Leghorn.

SPEVs samples are enriched for small RNAs and that almost all the miRNAs ranged between 18 and 35 nt in length and the peak value appeared at 22 nt (Figure 2). A total of 1077 miRNAs (851 known and 226 novel) ranging 17 to 28 nt were detected in the SPEVs samples of 4 breeds. From these, 542, 680, 656, and 665 were known miRNAs and 169, 167, 169, and 197 were novel miRNAs in BJY, DY, IV, and WL, respectively. Among the 4 breeds, we found that the top 10 abundant miRNA accounted for majority of total SPEVs miRNA reads, being 65%, 52%, 66%, and 61% for BJY, DY, IV, and WL, respectively (Figure 3). The most abundantly expressed 3 miRNAs in all breeds were gga-miR-10b-5p, gga-miR-10a-5p, and gga-miR-100-5p.

**Figure 4:**
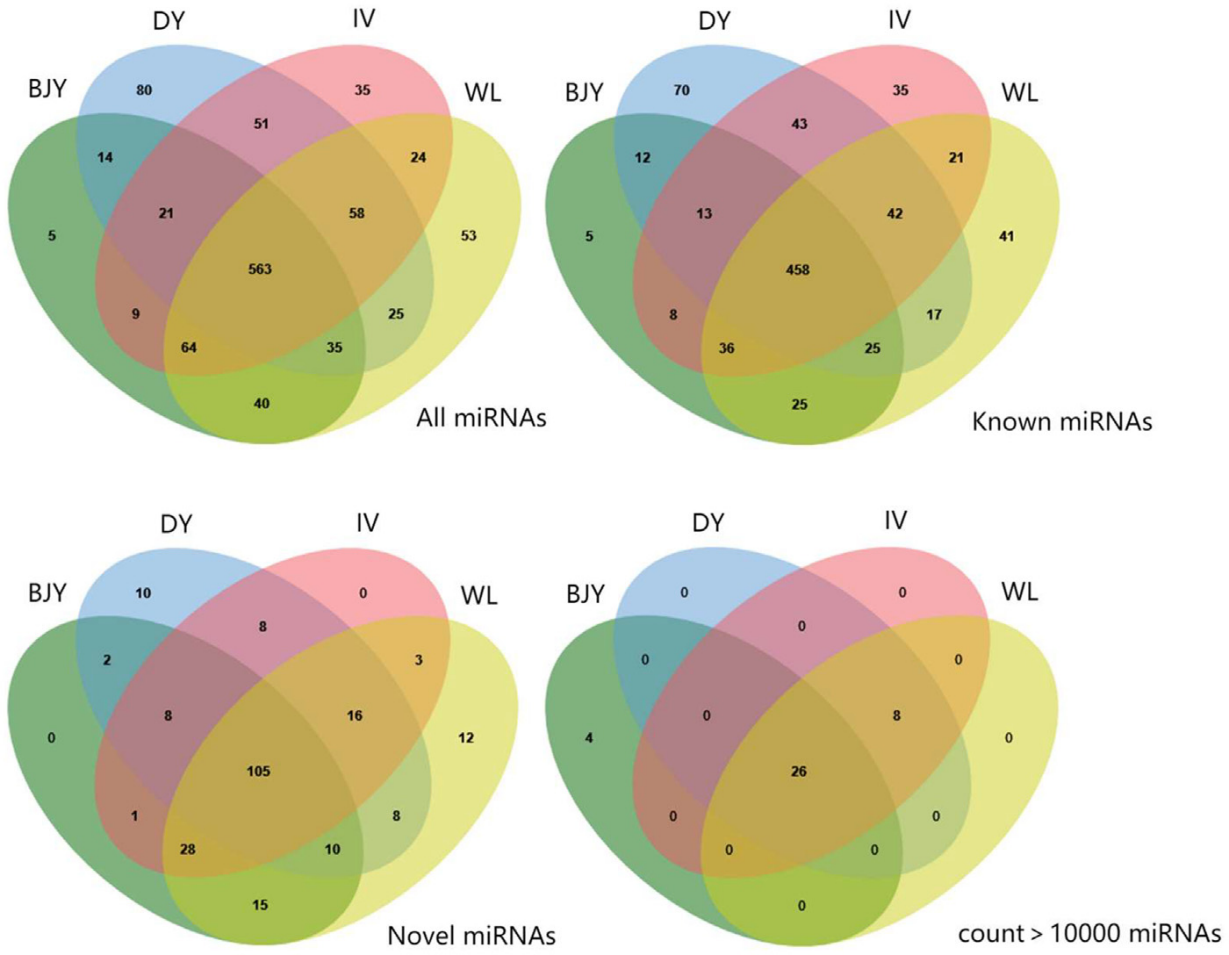
Venn diagram representing the distribution of all miRNAs, known miRNAs, novel miRNAs, and miRNAs with count >10,000 in seminal plasma extracellular vesicles of 4 chicken breeds. BJY, Beijing-You chicken; DY, Dwarf chicken; IV, Recessive white chicken; and WL, White Leghorn.

**Figure 5:**
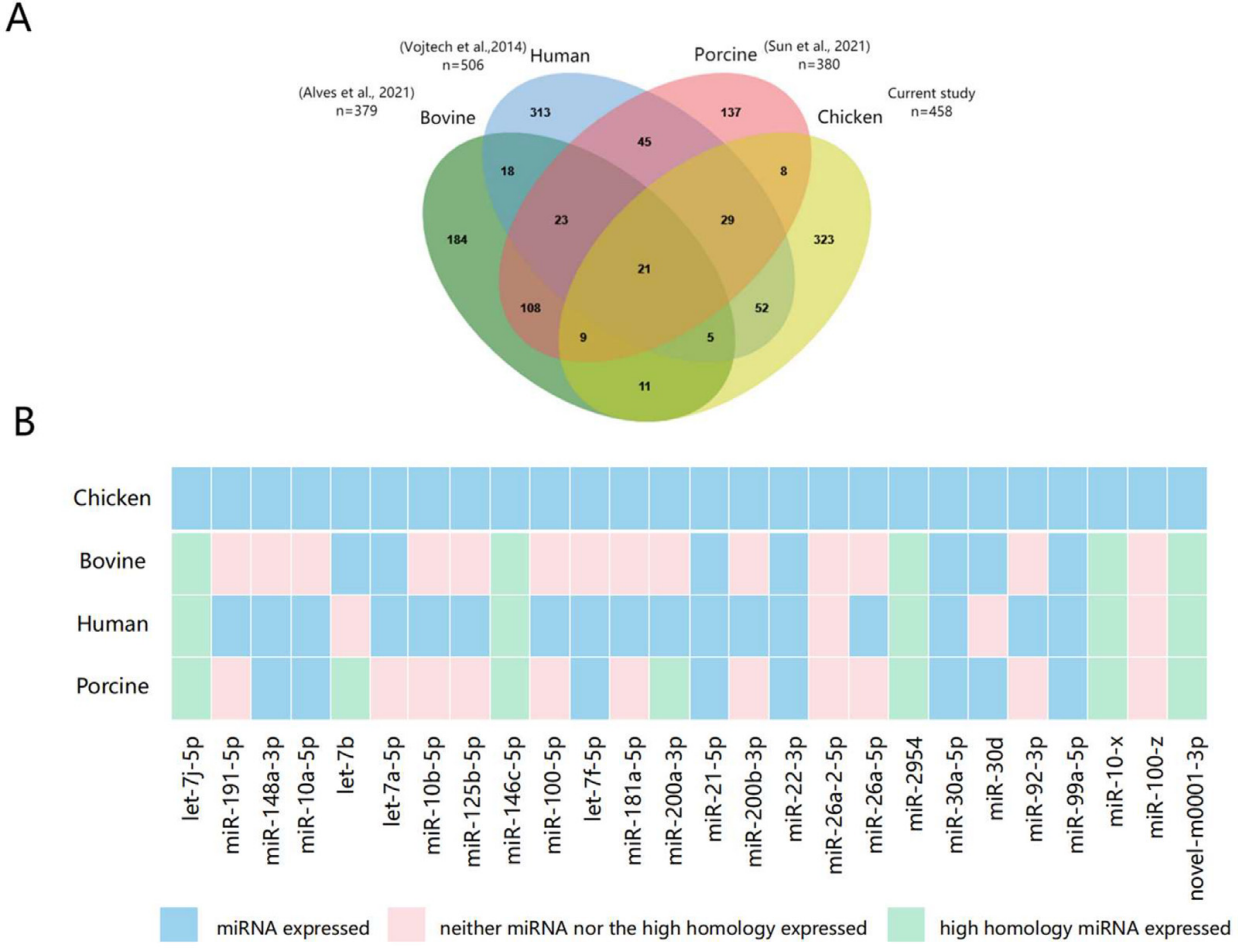
The expression of identified miRNAs of chicken seminal plasma extracellular vesicles in other species. (A) Comparison of the 458 known miRNAs identified in the current study with previously published data of bovine, porcine, and human (Alves et al., 2021; Sun et al., 2021; Vojtech et al., 2014). (B) Expression of 26 highly expressed miRNAs in chicken seminal plasma extracellular vesicles (count > 10,000) in other 3 species. Blue rectangular presents the same miRNA was expressed, pink one indicates high homologous miRNA was expressed, and green one represents the miRNA was not expressed.

Venn diagram results showed the overlap of miRNA in the 4 breeds. For the whole detected miRNA cluster, 563 miRNAs (458 known and 105 novel) were shared by all breeds, and 633 miRNAs were shared by at least 2 breeds. For the known miRNA cluster, 458 (42.5%) miRNAs were present in at least 2 breeds. For the novel miRNAs, 105 miRNAs were common to at least 2 breeds. In addition, 26 miRNAs were shared by all breeds with count >10,000 (Figure 4). All miRNAs with count >10,000 in the four breeds were shown in supplementary Table S1. A total of 21 common miRNAs were identified between different species including human, bovine and porcine, of which, 4 miRNAs (miR-21-5p, miR-22-3p, miR-30a-5p and miR-99a-5p) were among the aforementioned 26 abundantly expressed shared miRNAs (Figure 5). The 21 common miRNAs were listed in supplementary Table S2).

### Regulatory Function Analysis of miRNAs in SPEVs

For further investigating the potential function of SPEVs miRNAs, GO and KEGG pathway analyses was carried out for mRNA targets of the 563 common miRNAs from 4 breeds, and for mRNA targets of the 26 common miRNAs with count >10,000, respectively.

**Figure 6:**
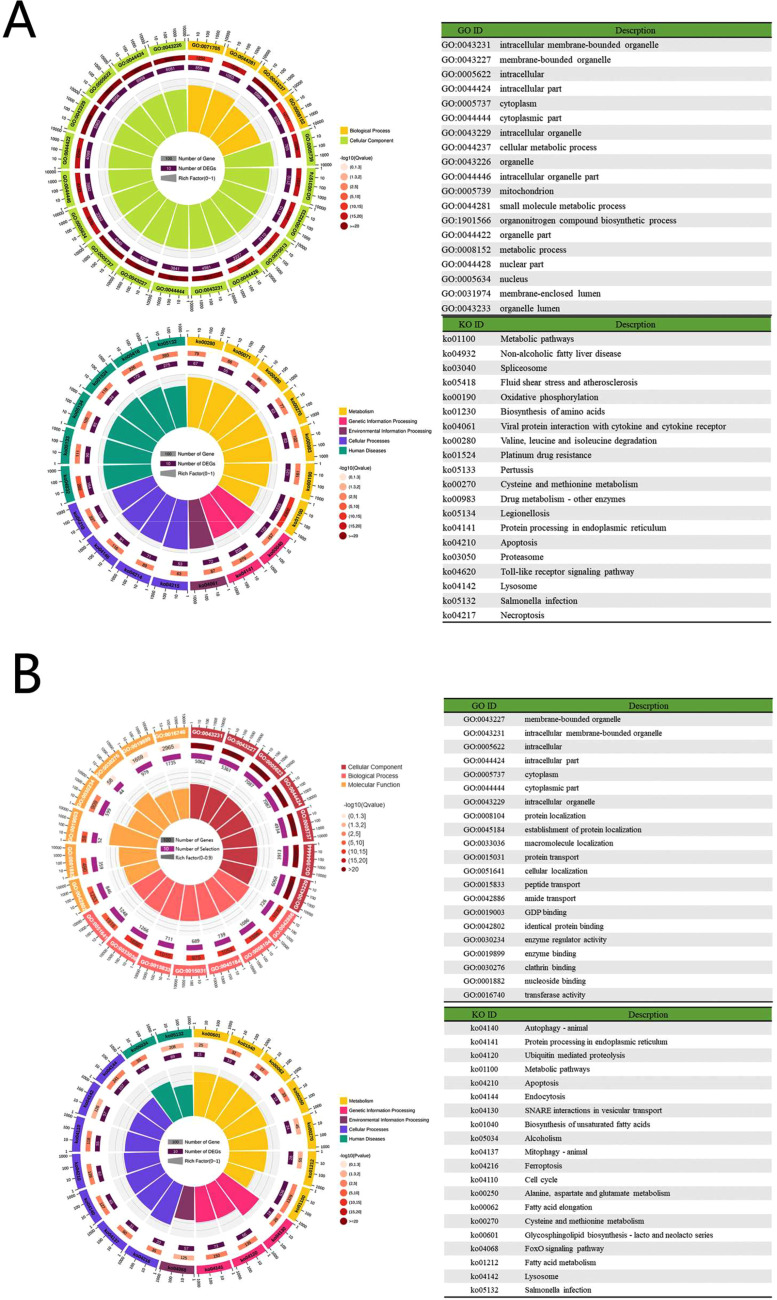
GO and KEGG analysis of target genes of SPEVs miRNAs in 4 chicken breeds. (A) GO classification and KEGG annotation of target genes of all common miRNAs in 4 chicken breeds. (B) GO classification and KEGG annotation of target genes of 26 common miRNAs with count >10,000 in 4 chicken breeds.

A total of 16,221 genes were identified as targets of the 563 common miRNAs. Among the different biological processes, the top 5 enrichments were cellular metabolic process, small molecule metabolic process, metabolic process, nitrogen compound transport, and regulation of protein metabolic process. The most dominant functional categories in the cellular component domain were membrane-bounded organelle, intracellular membrane-bounded organelle, intracellular, intracellular part, and cytoplasm. Meanwhile, within the molecular function category, purine nucleoside binding was the most significantly identified enriched term followed by nucleoside binding, ribonucleoside binding, purine ribonucleoside binding, and GTP binding. The identified target genes of 563 common miRNAs were plotted against the KEGG reference pathways, which mainly consisted of the following pathways: metabolic pathway, nonalcoholic fatty liver disease, and fluid shear stress and atherosclerosis, pertussis, and valine, leucine and isoleucine degradation, spliceosome, drug metabolism-other enzymes, Legionellosis, protein processing in endoplasmic reticulum, platinum drug resistance, and viral proteins interaction with cytokine and cytokine receptor, apoptosis, oxidative phosphorylation, *Salmonella* infection, peroxisome, apoptosis-multiple species, cysteine and methionine metabolism, fatty acid degradation, glutathione metabolism, and apoptosis-fly (Figure 6A).

A total of 7,570 genes were identified as targets of the abundantly expressed 26 common miRNAs. The most important functional categories enriched in the cell component domain were the same as 563 common miRNAs, protein localization, establishment of protein localization, macromolecule localization, and protein transport and cellular localization were highly enriched in biological processes. On the other hand, GO annotations in molecular function are mainly enriched in nucleoside bindings. Among the KEGG pathway of 563 common miRNAs and 26 common miRNAs with count >10,000, the shared enriched pathways were metabolic pathways, protein processing in endoplasmic reticulum, apoptosis, *Salmonella* infection, and cysteine and methionine metabolism (Figure 6B).

### Combined Analysis of SPEVs miRNA-Sperm mRNA and SPEVs miRNA-SST mRNA

**Figure 7:**
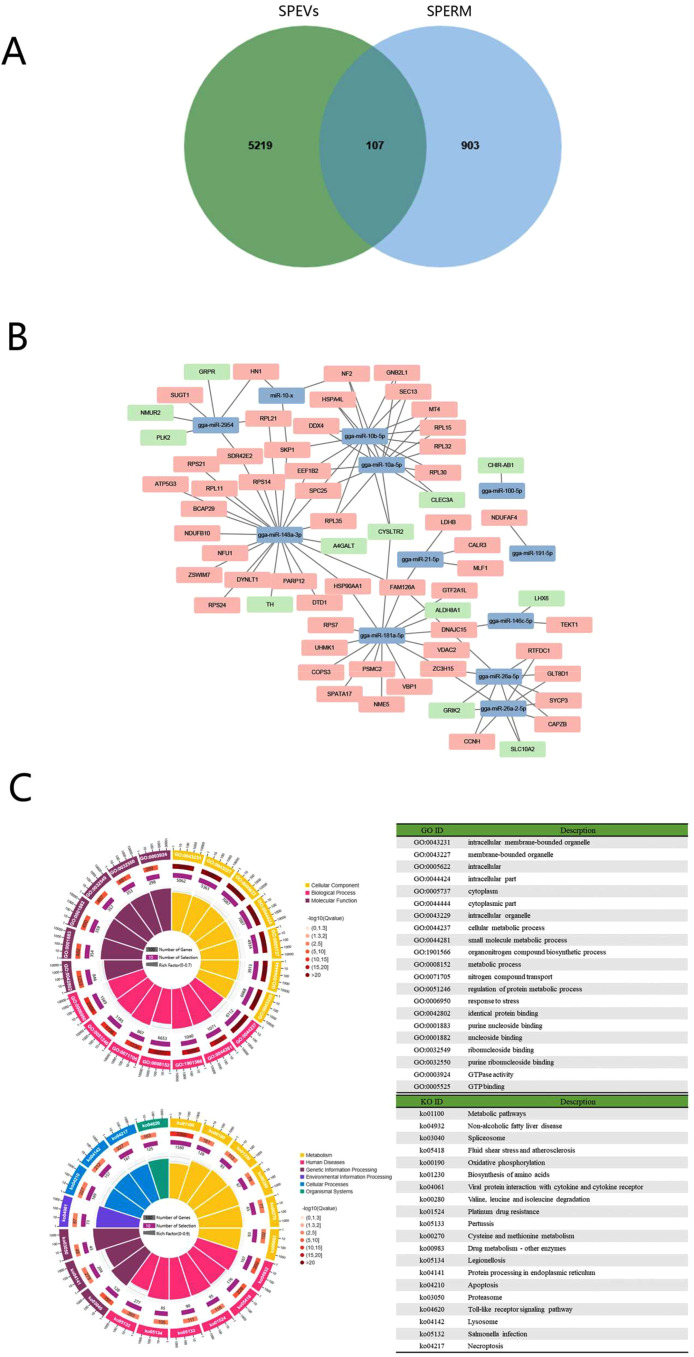
Analysis of SPEVs miRNA and sperm mRNA. (A) Venn diagrams representing the distribution of target genes mRNA of SPEVs and sperm mRNA. (B) Regulatory networks target genes mRNA of 26 miRNAs with count >10,000 and sperm mRNA. Green and pink represents the up- and down-regulated genes, respectively in the testicular difference analysis. (C) GO classification and KEGG annotation of 107 common genes between target genes of 26 common miRNA with count >10,000 and sperm mRNA.

Of the target genes of abundantly expressed 26 common miRNAs, 107 were also detected in the chicken sperm, one of the potential target cells of SPEVs miRNA (Figures 7A and 7B). The supplementary Table S3. The GO results of sperm mRNA combination analysis are more similar to the 563 miRNA results. KEGG analysis of those 107 selected target genes was mainly enriched to pathways such as metabolic pathways, oxidative phosphorylation, protein processing in endoplasmic reticulum, Toll-like receptor signaling pathway, and lysosome (Figure 7C).

**Figure 8:**
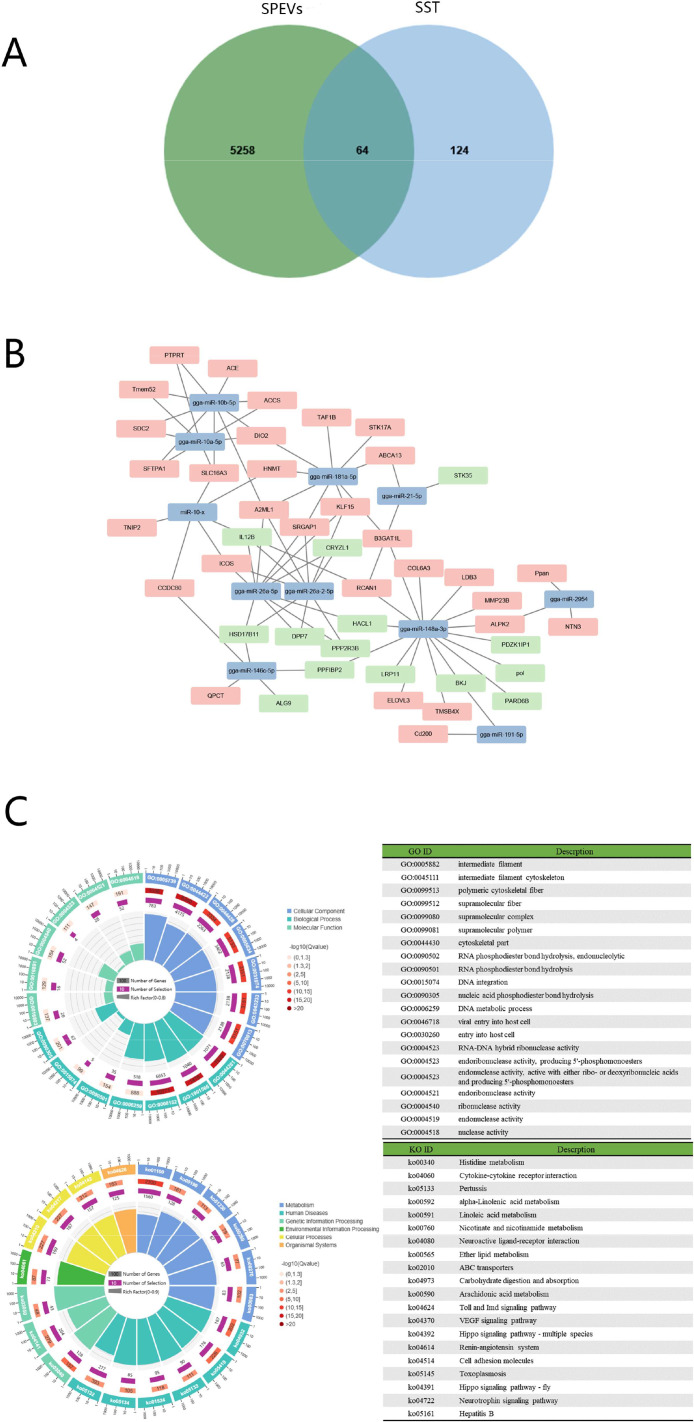
Analysis of SPEVs miRNA and female sperm storage tubules cells mRNA. (A) Venn diagrams representing the distribution of target genes mRNA of SPEVs and sperm storage tubules cells mRNA. (B) Regulatory networks target genes mRNA of 26 miRNAs with count >10,000 and sperm storage tubules cells mRNA. Green and pink represents the up- and down-regulated genes, respectively in the sperm storage tubules cells of females with high sperm storage ability. (C) GO classification and KEGG annotation of 64 common genes between target genes of 26 common miRNA with count >10,000 and sperm storage tubules cells mRNA.

Of the target genes of abundantly expressed 26 common miRNAs, 64 were also previously reported as crucial ones in chicken SST cells, which are also supposed to be the potential targets cells of SPEVs miRNAs (Figures 8A and 8B, Additional table 3). In GO and KEGG analysis of these selected targets, the top 5 biological processes enrichments were RNA phosphodiester bond hydrolysis, endonucleolytic, RNA cytoskeletal bond hydrolysis, DNA metabolic process, and viral entry into host cell and entry into host cell. The most dominant functional categories in the cellular component domain were intermediate filament, intermediate filament cytoskeleton, polymeric cytoskeletal fiber, and supramolecular fiber and supramolecular complex. Meanwhile, within the molecular function category, RNA–DNA hybrid ribonuclease activity, endoribonuclease activity, producing 5′-phosphomonoesters, endonuclease activity, active with either ribose deoxyribonucleic acids, producing 5′-phosphomonoesters, and endoribonuclease activity and ribonuclease activity were enriched. KEGG analysis of those 64 selected target genes was mainly enriched to biosynthesis of unsaturated fatty acids, fatty acid metabolism, histidine metabolism, ABC transporters, VEGF signaling pathway, hippo signaling pathway, and Toll and Imd signaling pathway (Figure 8C).

## DISCUSSION

In the present study, 4 chicken breeds were selected as experimental animals to reveal the miRNA expression profile of chicken SPEVs, which were further analyzed jointly with potential target cells including SST and sperm mRNA, to reveal the function of SPEVs in ruling of fertility.

SPEVs have been proved to exist in many species and almost all cell types and recent studies have mainly focused on the functions of their cargos including proteins, lipids, and nucleic acids (Machtinger et al., 2016). Here, the existence of chicken SPEVs was validated by TEM, NTA, and Western Blot at the same time. Previous studies have shown that dissociative miRNAs in semen participated in reproduction regulation (Chen et al., 2016; Yang et al., 2023). SPEVs-coupled miRNAs are more advantageous because their double-layer membrane structure protects the cargos against enzymatic degradation or phagocytosis/immune rejection (Chen et al., 2020). The 563 common miRNAs identified in this study were also enriched in metabolic pathways such as glycolysis and oxidative phosphorylation. These pathways are related to sperm ATP levels and hyperactivation motility, affecting sperm capacitation (Balbach et al., 2020; Tourmente et al., 2022) and sperm velocity (Tourmente and Roldan, 2015).

The gga-miR-21-5p, gga-miR-22-3p, gga-miR-30a-5p, and gga-miR-99a-5p were identified as highly conserved and highly expressed on chickens. It is reported that highly conserved miRNAs among species play more important regulatory roles as compared to those of not conserved (Lu et al., 2008). Proven by comparison between multiple species that SPEVs miRNAs are highly conserved across species. The miR-21-5p is reported to inhibit the vinculin gene that is associated with the constraining of sperm capacitation, and directly affects TLR4 signaling by promoting interleukin 10 and inhibiting interleukin 12 (Sheedy et al., 2010; Cui et al., 2017; Rodriguez-Martinez et al., 2021), demonstrating its association with immune regulation, which is important for sperm survival. The heat-sensitive miRNA miR-22-3p regulates potential heat-sensitive targets thioredoxin reductase 1 and protein kinase cAMP-dependent type II regulatory subunit beta in germ cells and is associated with heat stress in spermatozoa (Yadav et al., 2018). The miR-30a-5p reduces spermatogonial stem cells’ reactive oxygen species production and malondialdehyde levels, improving cell viability and antioxidant capacity (Corral-Vazquez et al., 2017; Khanlari et al., 2021). The voltage dependent anion channel 3, protein kinase cAMP-activated catalytic subunit alpha and erb-b2 receptor tyrosine kinase 4 were the target mRNAs of miR-99a-5p, which closely related to sperm count and sperm motility (Stanger et al., 2016; Liu et al., 2017; Sato et al., 2018).

The gga-miR-10b-5p, gga-miR-100-5p, and gga-miR-10a-5p are the most abundant 3 SPEVs miRNA detected in this study. MiR-10b, produced by Sertoli cells (Li et al., 2017), is also one of the top 10 extracellular vesicle miRNA in mice, human, boar, and bulls (Dlamini et al., 2023). Its over expression reduced spermatogonial apoptosis in primary spermatogonia (Gao et al., 2023). Over-expression of miR-100 promotes the proliferation of spermatogonial stem cells in vitro (Huang et al., 2017b). Another study in human detected significant under-expression of miR-10a-5p in spermatozoa from patients with teratozoospermia (Tomic et al., 2022). The miR-10a-5p is proposed to be related to the proliferation and differentiation of porcine spermatogonia (Chen et al., 2017; Keles et al., 2021). Let-7 is one of the most studied highly conserved miRNAs. Although family members of let-7 have been recognized as efficient tumor suppressors (Adams et al., 2015; Anastasiadou et al., 2018), they also exhibited fertility-related functions in recent studies. The high abundance of let-7 members was reported in the ovaries and testes of Portunus trituberculatus, indicating their essential roles in gonadal development (Meng et al., 2018). MiRNAs from the let-7 family were predicted to be involved in spermatogenesis and sperm motility in chickens and pigs by regulating high mobility group AT-hook 2 and phorbol-12-myristate-13-acetate-induced protein 1, respectively (Luo et al., 2015; Meng et al., 2018). Other than that, the identified miR-26a-5p was reported to affect the anti-apoptotic and survival-promoting functions of sperm cells by targeting phosphatase and tensin homolog in boar (Ma et al., 2018). The low levels of miR-26a-5p transcript have significant positive relationships with low sperm motility and high abnormal morphology (Dorostghoal et al., 2020).

The SPEVs miRNAs may participate in post-testicular modification of sperm in the endocrine (to distant targets cells) ways. Besides the function during the spermatogenesis, SPEVs may internalize their cargo in spermatozoa during the transport through the vas deferens and in female tract epithelia after the insemination to regulate their functional activity (Rodriguez-Martinez and Roca, 2022). This may be evidenced by studies showing that optimal fertility can be obtained when SPEV-coupled miRNAs from a fertile male, embeds spermatozoa maintaining their homeostasis, and that additional homologous/pools of semen plasma infused to the female genital tract resulted in better fertility in several species (Rodriguez-Martinez et al., 2021; Mahdavinezhad et al., 2022). On one hand, SSTs in the uterovaginal junction of oviduct are the sites for long term storage and release of sperm, and therefore crucial sperm functions, SST cells secrete exosomes and secrete them into the SST cavity in response to resident sperm. This process may be regulated by substances in sperm and semen (Huang et al., 2017a; Riou et al., 2020; Cordeiro et al., 2021b). On the other hand, the mRNAs in the spermatozoa have been proposed to have an effect on fertilization and embryo development (Steger et al., 2008; Singh et al., 2016).

The mRNA cluster in the sperm cells may play substantial roles in fertilization, embryo development, and even intergenerational epigenetics influence (Singh et al., 2016; Liang et al., 2022). The high abundant SPEV-coupled miRNAs also target 107 functional mRNAs in the sperm. The heat shock protein family A member 4 like and eukaryotic translation elongation factor 1 beta 2 targeted by gga-miR-10b-5p and gga-miR-10a-5p, respectively, were highly expressed in hypospermate individuals and were associated with embryonic development (Selvaraju et al., 2021; Mańkowska et al., 2022). The DEAD-box helicase 4 is a germline-specific gene and Transglutaminase 4, a prostate-specific gene used as a control gene, are also regulated by gga-miR-10b-5p and gga-miR-10a-5p (Kim et al., 2015; Yu et al., 2016a). The calreticulin 3, regulated by gga-miR-21-5p, affects male development by regulating the expression of ADAM metallopeptidase domain which is associated with sperm fertilization and sperm egg binding (Ikawa et al., 2011). The heat shock protein 90 alpha family class A member 1 regulated by gga-miR-148a-3p and gga-miR-181a-5p, is related to the heat stress conditions and antifreeze performance of sperm (Casas et al., 2010; Ramón et al., 2014).

In the present study, the high abundant SPEVs-coupled miRNAs were found to target 64 functionally important mRNAs in chicken SST cells. During the natural mating or artificial insemination, the semen plasma also enters the females’ oviduct. The SPEVs may regulate the gene expression of SST epithelial cells via transporting the miRNA cargos. KEGG analysis of those 64 selected target genes was mainly enriched to lipid metabolism (linoleic acid metabolism, alpha-linoleic acid metabolism, ether lipid metabolism, and arachidomic acid metabolism). It is reported that the superabundance of fatty acids secreted by SST cells had detrimental effects on sperm storage in the female reproductive tract (Wen et al., 2020). In addition, the enrichment of Toll and Imd signaling pathway, and cytokine-cytokine receptor interaction may indicate that the immune function of SST cells may be under regulation of SPEVs miRNAs. Antisperm immunity refers to the immune response caused by sperm entering the females as a foreign substance, which may be induced in the vagina of the oviduct (Zheng et al., 2001; Das et al., 2008). The SPEVs miRNAs may allow immune-mediated sperm protection until fertilization. The research in porcine proves that exosome induces endometrial immune and inflammatory response via C-C motif chemokine ligand 20 and alveolar macrophage-derived chemotactic factor-II regulation, which may facilitate sperm cells survival and activation, resulting in the establishment of appropriate uterine environment required for fertilization (Bai et al., 2018). The interleukin 12B, speculated to be targeted by miR-26a-5p here, was also low expressed in the hens of high fertility (Biscarini et al., 2010). Therefore, the SPEV-miRNAs may rule the fertility by participating the modulation of immune responses and fatty acid metabolism of female to tolerate the presence of foreign cells, the spermatozoa.

## CONCLUSION

In conclusion, chicken SPEVs-coupled miRNA was firstly identified with a total of 563 common ones among 4 chicken breeds. Some high expressed miRNAs were highly conserved in other species, suggesting their biological importance. Bioinformatics studies have shown that these miRNAs influence spermatogenesis and maturation. Taking sperm and SST cells as the potential target cells of SPEVs-coupled miRNAs, the combined analysis revealed that the miRNAs may rule the fertility by affecting the sperm maturation during the transport via the vas deferens and regulating the immune response and lipid metabolism in the females SST cells. Our findings may extend the understanding about the roles of miRNAs of SPEVs in sperm maturation, storage, and fertilization, and may help to develop new therapeutic strategies for male infertility and sperm storage in chickens.

## Appendix Supplementary materials

Image, application 1

Image, application 2

Image, application 3

## Footnotes

Supplementary material associated with this article can be found in the online version at doi:10.1016/j.psj.2023.103099.
